# Berry and Citrus Phenolic Compounds Inhibit Dipeptidyl Peptidase IV: Implications in Diabetes Management

**DOI:** 10.1155/2013/479505

**Published:** 2013-08-29

**Authors:** Junfeng Fan, Michelle H. Johnson, Mary Ann Lila, Gad Yousef, Elvira Gonzalez de Mejia

**Affiliations:** ^1^College of Bioscience and Biotechnology, Beijing Forestry University, Beijing 100083, China; ^2^Division of Nutritional Sciences, University of Illinois at Urbana-Champaign, Urbana, IL 61801, USA; ^3^Plants for Human Health Institute, NC Research Campus, North Carolina State University, Kannapolis, NC 28081, USA; ^4^Department of Food Science and Human Nutrition, University of Illinois at Urbana-Champaign, Drive, Urbana, IL 61801, USA

## Abstract

Beneficial health effects of fruits and vegetables in the diet have been attributed to their high flavonoid content. Dipeptidyl peptidase IV (DPP-IV) is a serine aminopeptidase that is a novel target for type 2 diabetes therapy due to its incretin hormone regulatory effects. In this study, well-characterized anthocyanins (ANC) isolated from berry wine blends and twenty-seven other phenolic compounds commonly present in citrus, berry, grape, and soybean, were individually investigated for their inhibitory effects on DPP-IV by using a luminescence assay and computational modeling. ANC from blueberry-blackberry wine blends strongly inhibited DPP-IV activity (IC_50_, 0.07 ± 0.02 to >300 **μ**M). Of the twenty-seven phenolics tested, the most potent DPP-IV inhibitors were resveratrol (IC_50_, 0.6 ± 0.4 nM), luteolin (0.12 ± 0.01 **μ**M), apigenin (0.14 ± 0.02 **μ**M), and flavone (0.17 ± 0.01 **μ**M), with IC_50_ values lower than diprotin A (4.21 ± 2.01 **μ**M), a reference standard inhibitory compound. Analyses of computational modeling showed that resveratrol and flavone were competitive inhibitors which could dock directly into all three active sites of DPP-IV, while luteolin and apigenin docked in a noncompetitive manner. Hydrogen bonding was the main binding mode of all tested phenolic compounds with DPP-IV. These results indicate that flavonoids, particularly luteolin, apigenin, and flavone, and the stilbenoid resveratrol can act as naturally occurring DPP-IV inhibitors.

## 1. Introduction

Type 2 diabetes is characterized by excessive blood glucose and insulin resistance due to an improper insulin response of the body to manage glucose from the diet [1]. Dipeptidyl peptidase IV (DPP-IV, EC 3.4.14.5), a serine peptidase, is one of the newest pharmaceutical targets for type 2 diabetes treatment [1]. On the other hand, incretin-based therapy has several potential sites of action for the treatment of type 2 diabetes ranging from increasing insulin secretion, reducing glucagon secretion, and regulating glucose control [2]. It is well known that glucagon-like peptide-1 (GLP-1) and glucose-dependent insulinotropic polypeptide (GIP) are major human incretin hormones that stimulate insulin release in a glucose-dependent manner in healthy individuals [3, 4]. However, DPP-IV rapidly transforms these two gut incretin hormones after secretion by cleavage of the penultimate proline or alanine at N-terminus, and thus forms their inactive metabolites [5–7]. Both hormones have very short half-lives (approximately 2 min) due to the rapid degradation by DPP-IV [8]. Among the several peptide substrates of DPP-IV, GLP-1 is one of the well-characterized physiological and pharmacological substrates of the enzyme. GLP-1, which is secreted in a nutrient-dependent manner, stimulates glucose-dependent insulin secretion and regulates glycemia. However, the actions of GLP-1 do not last long due to degradation by DPP-IV. For this reason, DPP-IV inhibition is expected to result in elevated plasma insulin levels by inhibiting the degradation of active GLP-1 after oral glucose intake. This in turn leads to the suppression of blood glucose elevation. Therefore, development of DPP-IV inhibitors is being actively conducted worldwide, and control of blood glucose levels by enhancement of GLP-1 action is a new option for the treatment of diabetes. 

In recent years, protein-ligand docking has become a powerful tool for drug development, and is also a method to be able to identify binding modes with high accuracy. For DPP-IV, computational docking analyses have been commonly used for designing inhibitors [9], screening of potential inhibitors [10], and explaining the differences in activity of drugs with different structures [11]. However, most of the previously investigated inhibitors of DPP-IV have been synthetically derived. As for naturally occurring flavonoids, the binding modes with DPP-IV are still not yet established.

Phenolic compounds, such as flavonoids, widely abundant in fruits and vegetables, have been suggested as important compounds for diabetes reduction [9, 10]. However, so far only a few phenolic compounds have been investigated to inhibit DPP-IV activity. These include procyanidin from grape seeds [12] and naringin from orange peel [13]. Therefore, it is necessary to further elucidate the modulating effect on DPP-IV activity of phenolic compounds from other natural sources. 

In epidemiological studies, berries were the most important contributors to lowering risk for type 2 diabetes [14]. Additionally, an inverse relationship between intake of flavonoids, specifically those from berries, and risk of type 2 diabetes was found [15]. However, there is lack of evidence for the role of specific phenolics in clinical trials, and there is not yet sufficient data to confirm that anthocyanins have a protective effect against the risk of type 2 diabetes [16]. Additionally, anthocyanins found in berries have been found to have a beneficial effect on glucose metabolism; however, stronger scientific evidence is needed.

Anthocyanins (ANC) from blueberry-blackberry wine blends have been evaluated for DPP-IV and carbohydrate-utilizing enzymes inhibitor studies in our laboratory, and they have exhibited potent DPP-IV and *α*-glucosidase inhibitory activities [17]. Thus, the aim of the present study was to further characterize the ANC-rich fractions from blueberry-blackberry wine blends by HPLC and analyze their DPP-IV inhibitory effect *in vitro*. Furthermore, a variety of other phenolic compounds commonly present in berries, citrus, and other plant foods were studied for their DPP-IV inhibitory activity. We hypothesized that berry and citrus phenolics could bind to the active sites of DPP-IV, thus inhibiting DPP-IV enzyme activity. For the most potent compounds, kinetic and computational docking analyses were used to elucidate the binding modes with the DPP-IV enzyme. 

## 2. Materials and Methods

### 2.1. Materials

Wines were produced from highbush blueberry (*Vaccinium corymbosum*) cultivars Blue Chip, Bluecrop, Blue Haven, Blue Jay, Blueray, Bluetta, Collins, Coville, Darrow, Earliblue, Elliot, Jersey, Late Blue, and Spartan and blackberry (*Rubus fruticosus*) cultivars A-1937, A-2215, A-2241 Natchez, A-2315, APF 27, APF 40, APF 41, and Prime Jan, collected from Dixon Springs Agricultural Center in Simpson, IL, USA during the ripening season of 2010. Blueberry wine and blackberry wine were separately fermented using *Saccharomyces bayanus* as previously described [17]. After the fermentation, blends ranging from 100% blueberry to 100% blackberry were made using room temperature fermented wines. Blends were prepared with different ratios of % *blueberry * : % *blackberry*. The ratios were 100 : 0, 75 : 25, 25 : 75, and 0 : 100 of blueberry: blackberry wine blends, respectively.

All solvents used for phenolic extraction were HPLC-grade and were purchased from Fisher Scientific (Pittsburg, PA). Amberlite XAD-7 was purchased from Sigma-Aldrich (St. Louis, MO). Sephadex LH-20 was purchased from GE Life Sciences (Buckinghamshire, UK). Porcine kidney DPP-IV enzyme (88% sequence homology with human; both are homodimers with a subunit molecular mass of ~30 kDa) and diprotin A were purchased from Sigma-Aldrich. DPP-IV Glo^TM^ Protease Assay kits were purchased from Promega (Madison, WI). Flavonoids with high purities that were purchased from Sigma-Aldrich included luteolin (>98%), apigenin (>95%), quercetin (>98%), kaempferol (>97%), rutin hydrate (>94%), naringenin (>95%), neohesperidin (>90%), flavone (>97%), naringin (>90%), hesperidin (>80%), cyanidin-3-glucoside (>95%), cyanidin (>95%), malvidin (>95%), resveratrol (>99%), protocatechuic acid (>97%), catechin (>98%), epicatechin (>90%), epigallocatechin gallate (EGCG, >95%), gallic acid (>97.5%), caffeic acid (>98%), and chlorogenic acid (>95%). Hesperetin (>95%) was purchased from Sigma-Aldrich (Wicklow, Ireland) and limonin (>90%) from MP BioMedicals (Solon, OH). Narirutin (>93.9%) and eriocitrin (>97.4%) were purchased from Chromadex (Irvine, CA). Genistein (>90%) and genistin (>90%) were kindly donated by Dr. Mark Berhow, USDA. All other reagents were of analytical grade.

### 2.2. Phenolic Extraction and Preparation of ANC Fractions

Phenolic extraction and preparation of ANC fractions were conducted as previously described [17]. Briefly, each wine was firstly acidified, dealcoholized, and then mixed with amberlite XAD-7 resin to remove sugars and phenolic acids. After nonpolar compounds were further removed from the crude polyphenolics, the polar eluate was loaded onto a Sephadex LH-20 column to generate ANC-enriched fractions. With an isocratic elution using water : methanol (80 : 20, containing 0.1% TFA) and then 50% methanol, five anthocyanin-rich fractions (ANC 1–5) were obtained. ANC 2–5 from each blend of blueberry and blackberry were analyzed by HPLC to determine their ANC composition.

### 2.3. Anthocyanin Analysis

ANC analyses were conducted as previously published [17] using a 1200 HPLC (Agilent Technologies, Santa Clara, CA) with a Supelcosil LC-18 RP column (250 × 4.6 mm, 5 *μ*M) (Supelco, Bellefonte, PA). ANC were detected at 520 nm using a diode array detector (DAD). Specific anthocyanins were identified based on comparison to our previously published data [18, 19]. A previously well-characterized blueberry extract [19] was included with each sample run to verify compound separation and identification. Using the peak areas as measured by HPLC at 520 nm, total ANC were quantified from a standard curve generated from 0.125, 0.25, 0.5, and 1.0 mg/mL of cyanidin-3-glucoside (C3G) and ANC amounts are presented as C3G equivalents.

### 2.4. DPP-IV Inhibition

Measurement of the activity and potential inhibition of DPP-IV, a type II membrane glycoprotein, was done using the DPP-IV Glo^TM^ Protease Assay following the manufacture's protocol (Promega, Madison, WI). Briefly, 50 *μ*L of DPP-IV Glo^TM^ reagent was added to a white-walled 96-well plate containing 50 *μ*L of blank, positive control, or treatment. The blank contained the vehicle only while positive control contained the vehicle and purified DPP-IV enzyme (at a final concentration of 1 ng/mL). Treatments used were enriched ANC fractions (0.5, 5, 20, and 40 *μ*g/mL), phenolic compounds (0.5, 5, 20 and 40 *μ*g/mL) or known inhibitor, diprotin A (1, 2, 12, 24, 125, and 250 *μ*M), and the purified DPP-IV enzyme at a final concentration of 1 ng/mL. The content of the wells was gently mixed using an Ultra Microplate Reader (Biotek Instruments, Winooski, VT) at medium intensity for 4 s. DPP-IV cleavage of the provided Gly-Pro-amino methyl coumarin (AMC) substrate generated a luminescent signal by luciferase reaction, with the amount of DPP-IV enzyme available to bind Gly-Pro-AMC proportional to relative light units (RLU) produced. This signal in RLU was measured after 30 min in the Ultra Microplate Reader and then compared to the blank. Diprotin A linear standard curve (*y* = 41.936*x* + 27.294, *R* ^2^ = 0.91), where *y* was the % inhibitory activity of diprotin A and *x*  was the log_10_ of the concentration (*μ*M) of known inhibitor diprotin A, was used to calculate IC_50_ value: the concentration needed to decrease the activity of the enzyme by 50% of its original activity. IC_50_ values were calculated based on the molecular mass of each compound or C3G as the equivalent for ANC-enriched fractions.

### 2.5. Inhibitory Kinetics Study

Porcine kidney DPP-IV activity was measured at various concentrations of three flavonoids (5 and 10 mg/mL for luteolin, apigenin, and flavone; and 0.25 and 0.5 mg/mL for resveratrol). Each concentration was evaluated in the presence of various concentrations of Gly-Pro-AMC (0–60 *μ*M). DPP-IV activity was measured using the DPP-IV Glo Protease Assay as mentioned above. The inhibition pattern was evaluated utilizing the Lineweaver-Burk plot. Enzyme-inhibition constant *K*_*i*_ was determined by plotting the reciprocal of the initial luminescence versus the reciprocal of the initial substrate concentration. 

### 2.6. Molecular Modeling and Computational Docking Study

The DPP-IV enzyme exists as a dimer in the crystal form, and each monomer consists of 726 amino acids [20]. The docking studies were conducted with the monomeric unit of the enzyme, as the active site of the enzyme resides deep within each monomer of the receptor protein and not on the enzyme surface [21]. The molecular docking analysis of flavonoids was carried out using AUTODOCK 4.2 (CCDC, UK; http://www.ccdc.cam.ac.uk/products/csd/) [22]. The crystal structure of the DPP-IV enzyme (Protein Data Bank (PDB) ID: 2I03) was obtained from the protein data bank (http://www.rcsb.org/pdb), and the protein structure was prepared using Accelrys Discovery Studio 3.5 program (Accelrys Software Inc., San Diego, CA). For the computational docking study, the energies of diprotin A and flavonoids were minimized by applying a CHARM22 force field, using the Accelrys Discovery Studio 3.5 program. After removing water molecules and adding all the hydrogen atoms, Gasteiger-Hückle charges were assigned to the enzyme. The ligand conformers were treated as flexible and protein structures were treated as rigid during the docking process. The docking was carried for 100 genetic algorithm runs, which was optimum to validate the crystal structure of the ligand. Most of the other genetic algorithm parameters such as the population size were maintained at their default values. The best docking results were considered to be the conformation having the lowest binding energy (Δ*G*) using:

(1) ΔG=ΔG  (intermolecular)+ΔG  (internal)+ΔG  (tor)−ΔG  (unbound  extended),

where Δ*G* (intermolecular) denotes the sum (kcal/mol) of Van der Waals energy, hydrogen bond energy, electrostatic energy, and desolvation energy; Δ*G* (internal) is the final total internal energy (kcal/mol); Δ*G* (tor) denotes torsional free energy (kcal/mol); and Δ*G* (unbound extended) is the unbound system's energy (kcal/mol).

In the context of Autodocking, inhibition constant (*K*_*i*_) is directly related to the binding energy:

(2) $$\begin{array}{c} K_{i}=e^{\left[\Delta G/(RT)\right]}, \end{array}$$

where *e* is the base number of natural logarithm (approximately equals 2.72), *R* is the gas constant (kcal/mol), and *T* is the absolute temperature. Smaller *K*_*i*_ and more negative Δ*G* mean tighter binding.

### 2.7. Statistical Analyses

Data were expressed as means of independent duplicates with at least three replicates. The dose-response analysis of each compound on DPP-IV activity was performed using nonlinear or linear regression (curve fit) using EXCEL Microsoft (e.g., see Supplementary Information available online at http://dx.doi.org/10.1155/2013/479505). Statistical analysis was conducted using the proc GLM procedures of SAS version 9.3 (SAS Inst. Inc., Cary, NC, 2009). Group mean comparisons were conducted using Duncan means and were considered to be significant at *P* < 0.05 based on the least significant differences (LSD) from one-way analysis of variance (ANOVA) with alpha = 0.05. Correlations were made using Pearson's correlation values with *P* < 0.05.

## 3. Results

### 3.1. Blackberry Wine Presented High Concentrations of Delphinidin-3-arabinoside

Anthocyanin relative distributions in the extracts of blueberry-blackberry wine blends are shown in Table 1. Chromatographic analyses revealed up to seventeen ANC present in blueberry-blackberry wine blends. Malvidin-3-galactoside and cyanidin-3-glucoside were the main ANC present in the blueberry wine, while delphinidin-3-arabinoside was the predominant ANC present in the blackberry wine. Total ANC ranged from 1653.8 mg C3G equivalents/L for blueberry wine to 3267.8 mg C3G equivalents/L for blackberry wine. It was also observed that there was an obvious difference between ANC amounts of different fractions generated as ANC 2–5.

### 3.2. Anthocyanins from Blackberry Wine Potently Inhibited DPP-IV

ANC-enriched fractions (ANC 1–5) isolated from blueberry-blackberry wine blends were analyzed for their DPP-IV inhibitory effect.  Table 2 shows the IC_50_ values of ANC from blueberry-blackberry wine blends needed to inhibit DPP-IV enzyme. Compared to a standard curve of diprotin A (IC_50_, 4.21 ± 2.01 *μ*M), a known DPP-IV inhibitor with an Ile-Pro-Ile sequence, ANC 2–5 tested at concentrations of 0.5, 5, 20, and 40 *μ*M in C3G equivalents obtained from each blend had IC_50_ values ranging from 2.64 ± 1.40 *μ*M in ANC 2 from blueberry wine to 0.07 ± 0.02 *μ*M in ANC 3 from blackberry wine (Table 2).  Table 2 also shows that ANC from blackberry wine were the most effective of the blends at reducing the activity of DPP-IV (with IC_50_ values of no more than 0.22 *μ*M C3G).

### 3.3. Resveratrol, a Stilbenoid, Luteolin, Apigenin, and Flavone, Flavonoids Commonly Present in Fruits, Have Strong DPP-IV Inhibitory Activity

Twenty-seven phenolic compounds commonly present in citrus, berries, grape, soybeans, and other plants were tested for DPP-IV inhibitory effect (Table 3). Sixteen phenolic compounds demonstrated DPP-IV inhibitory activity with IC_50_ values ranging from 0.6 ± 0.4 nM (resveratrol) to 10.36 ± 0.09 *μ*M (eriocitrin). Eleven compounds did not have DPP-IV inhibitory activity including rutin, narirutin, naringin, hesperidin, limonin, neohesperidin, genistin, catechin, epicatechin, chlorogenic acid, and protocatechuic acid (data not shown). 

Of the sixteen effective phenolic compounds, three had IC_50_ values higher than diprotin A (4.21 ± 2.01 *μ*M) including eriocitrin (IC_50_ value of 10.36 ± 0.09 *μ*M), EGCG (10.21 ± 0.75 *μ*M), and gallic acid (4.65  ±  0.1 *μ*M). However, IC_50_ values of the other thirteen compounds were lower than that of diprotin A, indicating that less of these compounds was needed to inhibit DPP-IV. These thirteen phenolics could be divided into three categories according to the results of statistical differences on their DPP-IV inhibitory effect: less active with high IC_50_ values (1.31–3.37 *μ*M), intermediate activity with IC_50_ values of 0.24–0.74 *μ*M, and very high activity with low IC_50_ values (0.0006–0.17 *μ*M). The phenolic compounds with high IC_50_ values were cyanidin, quercetin, and caffeic acid; the ones with intermediate activity were naringenin, hesperetin, cyanidin-3-glucoside, kaempferol, and malvidin. The four phenolics with very high activity included resveratrol, luteolin, apigenin, and flavone. IC_50_ value of resveratrol had the highest DPP-IV inhibitory activity among all of the compounds tested (*P* < 0.05).

### 3.4. Resveratrol and Flavone Inhibited DPP-IV Activity in a Competitive Manner, While Luteolin and Apigenin Inhibited Noncompetitively

**Figure 1:**
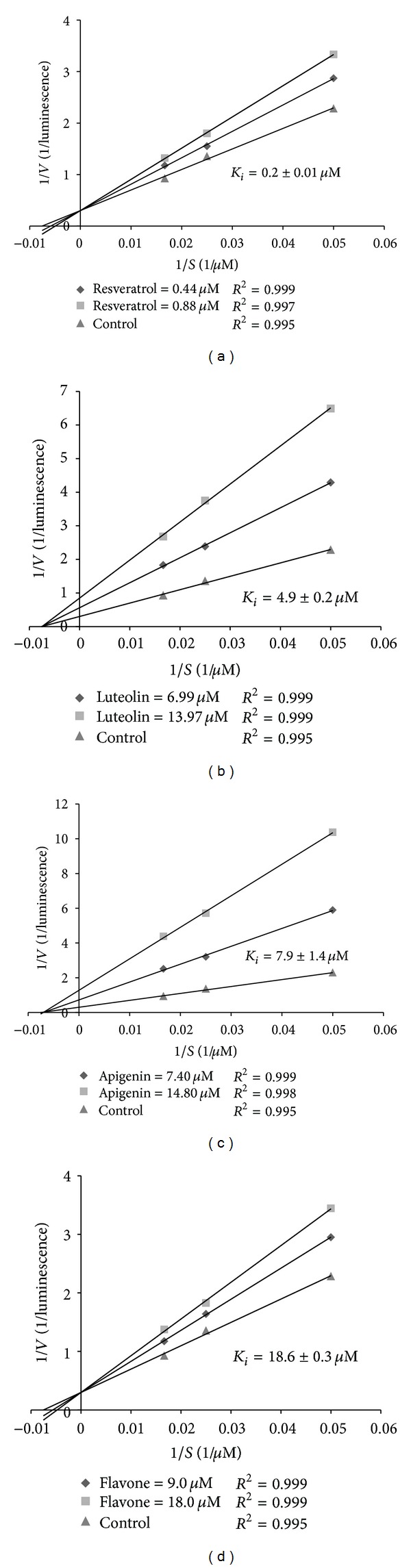
Inhibition kinetics of porcine dipeptidyl peptidase-IV (DPP-IV) by resveratrol (a), luteolin (b), apigenin (c), and flavone (d). Different concentrations of the flavonoids (0, 5, and 10 *μ*g/mL for luteolin, apigenin, and flavone and 0, 0.25, and 0.5 *μ*g/mL for resveratrol) were incubated in the presence of various concentrations of Gly-Pro-AMC (0–60 *μ*M) as substrate. Initial rates of the reaction were measured, and the results are expressed as a Lineweaver-Burk plot. Data are expressed as the mean of four independent experiments.

To examine whether the most potent phenolic compounds, resveratrol, luteolin, apigenin and flavone, inhibited DPP-IV through interaction with the active site of the enzyme, we tested the enzyme kinetics. The inhibitory manner of the flavonoids was determined through generating a Lineweaver-Burk plot (Figure 1). As noted in Figures 1(a) and 1(d), both the slope and the *x*-intercept were changed by the addition of inhibitors, but there was no effect on the *y*-intercept. This is the definition of linear competitive inhibition. Therefore, resveratrol (Figure 1(a)) and flavone (Figure 1(d)) inhibited DPP-IV activity in a competitive manner. The *K*_*i*_ values were calculated to be 0.2 ± 0.01 *μ*M for resveratrol and 18.6 ± 0.3 *μ*M for flavone. As for luteolin and apigenin (Figures 1(b) and 1(c)), both the slope and the *y*-intercept were changed by the added inhibitors, but there was no effect on the *x*-intercept. Therefore, luteolin and apigenin noncompetitively inhibited the enzyme, with *K*_*i*_ values at 4.9 ± 0.2 *μ*M and 7.9 ± 1.4 *μ*M, respectively.

### 3.5. Diprotin A and Natural Phenolic Compounds Inhibit DPP-IV Activity by Binding Tightly into the Active Site of the Enzyme

**Figure 2:**
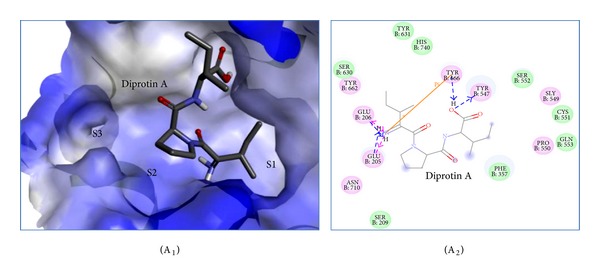
Key interactions of diprotin A (A_1_, A_2_) with active sites of DPP-IV enzyme. Binding of diprotin A (A_1_, grey) in the DPP-IV active site is indicated (surface view: blue), wherein it interacts closely with key residues of active sites S1, S2, and S3. Residues with pink circles indicate hydrogen bond, or ionic or polar interactions; residues with green circles indicate Van der Waals interactions. The arrows indicate hydrogen bonds to side chain residues in blue and backbone residues in green.

**Figure 3:**
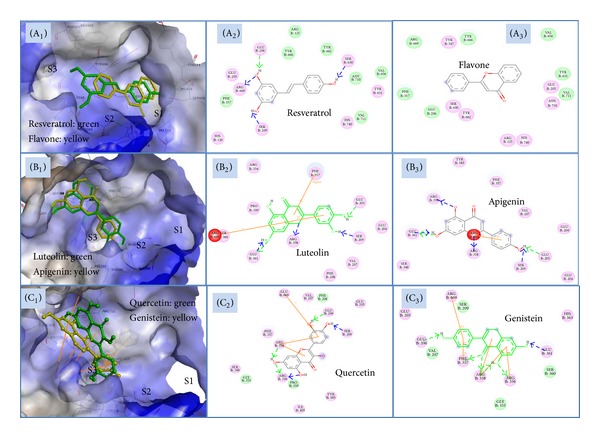
Key interactions of resveratrol (A_1_, A_2_), flavone (A_1_, A_3_), luteolin (B_1_, B_2_), apigenin (B_1_, B_3_), quercetin (C_1_, C_2_), and genistein (C_1_, C_3_) with active sites of DPP-IV enzyme. Binding pose of resveratrol (A_1_, green) and flavone (A_2_, yellow) in the DPP-IV active site is indicated (surface view: blue), wherein two compounds interact closely with key residues of active sites S1, S2, and S3. Binding pose of luteolin (B_1_, green), apigenin (B_1_, yellow), quercetin (C_1_, green) and genistein (C_1_, yellow) in the DPP-IV binding site is indicated, wherein these flavonoids interact closely with the key residues of sites S2, and S3. Residues with pink circles indicate hydrogen-bond, or ionic or polar interactions, residues with green circles indicate Van der Waals interactions. The arrows indicate hydrogen bonds to side chain residues in blue and backbone residues in green.

Binding pose of diprotin A, resveratrol and flavone in the DPP-IV active site is indicated in Figures 2 and 3, showing that these three compounds interact closely with key residues of sites S1, S2 and S3 within the active pocket.

Diprotin A is a potent DPP-IV inhibitor with Ile-Pro-Ile sequence commonly used as a reference compound.  Figure 2 shows the binding mode of diprotin A with DPP-IV. The binding site of diprotin A is located at the S1, S2 and S3 sites (Figure 2(A_1_)). In the S2 site (Figure 2(A_2_)), the N-terminal amino group of diprotin A is hydrogen-bonded to the carboxyl oxygens of two Glu residues (Glu205 and Glu206). Furthermore, the N-terminal amino group forms a *π* interaction to the Tyr666. The carbonyl oxygen of Ile-1 of diprotin A forms an electrostatic interaction with Tyr662, Arg125, and Asn710 residues. Pro-2 of diprotin A is located in the S1 site and forms a hydrophobic interaction with the phenol rings of Tyr666, and Tyr547. The carbonyl oxygen of Ile-3 of diprotin A also forms double hydrogen bonds to Tyr547 and Tyr666. In the S3 site, Van der Waals interactions are also seen between diprotin A and Ser209 and Phe357 residues of DPP-IV. These observations agree with the reported results obtained from X-ray crystal structure complex of DPP-IV and diprotin A [20].

 The overlay of binding poses of resveratrol (green) and flavone (yellow) in the DPP-IV active site is shown in Figure 3(A). As observed in Figure 3(A_1_), resveratrol and flavone dock very well into all three active sites S1, S2, and S3 of DPP-IV. Resveratrol showed hydrogen bonding of 4′-OH-, 3′-OH-, and 5′-OH-group with hydroxyl of side chain of Ser630 (S1 pocket) and Ser209 (S3 pocket). Hydrogen bonds were also seen between 5′-OH of resveratrol, NH_2_-group of side chain of Arg 669 residues, and C=O groups of side chains of Glu206 (S2 pocket) (Figure 3(B_2_)). At the same time, electrostatic interactions were also observed between resveratrol and S1 pocket (His740, Tyr631, Ser630, His125), S2 pocket (Glu205, Glu206), S3 pocket (Ser209), and Arg669 of DPP-IV. 

No hydrogen bonds were seen between flavone and amino acids in the pockets of DPP-IV (Figure 3(A_3_)). However, electrostatic interactions between flavone and amino acid residues in S1 pocket (Tyr547, Ser630, Asn710, and His740), S2 pocket (Arg125, Glu205), and Van der Waals interactions between flavone and amino acid residues in S1 pocket (Tyr631, Val656, Tyr666, Val711), S2 pocket (Glu206) and S3 pocket (Phe357), allowed flavone to anchor in the active sites of DPP-IV.

The overlay of binding poses of luteolin (green) and apigenin (yellow) in the DPP-IV active site is also shown in Figure 3(B). As shown in Figure 3(B_1_), luteolin and apigenin had almost identical binding modes with the active sites of DPP-IV with each having ring B and C docked into sites S2 and S3. Three common features of binding with DPP-IV exist between both flavonoids (Figures 3(B_2_) and 3(B_3_)). Firstly, hydrogen bonds and *π*-interactions played important roles in docking both the flavonoids into the active pockets S2 and S3 of DPP-IV enzyme. In the S2 pocket, the B ring 5′-hydroxyl of luteolin formed a hydrogen bond with hydroxyl group of side chain of Ser209 (S3 pocket), while the 4′-hydroxyl group on B ring of apigenin forms a similar hydrogen bond within the S3 pocket. Luteolin also showed H-bonding by B ring 4′-hydroxyl with C=O groups of side chains of Glu205 (S2 pocket). Secondly, in the S3 pocket, both compounds formed a hydrogen bond of the C ring 1′-oxygen with the NH of Arg358's guanidine side chain. H-bonding of the A ring 8′-hydroxyl with C=O groups of side chains of Glu361 favors strong binding of both flavonoids to the DPP-IV active site. A third common feature of both flavonoids was shown by *π*-cation interactions of ring B and the NH_2_ of Arg669. Additional features of each flavonoid added to their unique docking within the active site of DPP-IV. A hydrogen bond between the A ring 5′-hydroxyl of apigenin and the NH of Arg361's guanidine side chain also enhanced the docking of apigenin and DPP-IV. For luteolin, a *π*-sigma interaction was also seen between the side chain of Phe357 and C ring of luteolin (S3 pocket). 

Quercetin and genistein had a comparable binding position to luteolin and apigenin (Figure 3(c)). Hydroxyl groups in A and B rings were also important for quercetin and genistein to bind into the S2 and S3 sites (Figures 3(C_2_) and 3(C_3_)). At the same time, *π*-interactions between these flavonoids and Arg358 and Arg669 also contributed to the tethering of the two flavonoids to the active sites. All hydrogen bonds formed between phenolic compounds and DPP-IV are indicated in Table 3.

The binding energies obtained by computational docking analyses were compared among the compounds tested (Table 3). Gallic acid had the highest binding energy (−3.96 kcal/mol), while diprotin A had the lowest binding energy (−7.31 kcal/mol). IC_50_ values of the phenolic compounds that were found to inhibit DPP-IV activity correlated with their binding energies (*r* = 0.67, *P* < 0.05). Both a lower IC_50_ value and lower binding energy indicate stronger inhibitory potency. The inhibition constant (*K*_*i*_) obtained by computational docking analyses is also shown in Table 3. The *K*_*i*_ values of these phenolic compounds varied from 1.25 *μ*M for gallic acid to 604.91 *μ*M for EGCG. A highly significant correlation existed between *K*_*i*_ values and IC_50_ values (*r* = 0.82, *P* = 0.0002). Significant correlations were also found between *K*_*i*_ values and binding energies (*r* = 0.56, *P* < 0.05), and between *K*_*i*_ values and number of hydroxyl group (*r* = 0.56, *P* < 0.05).

## 4. Discussion and Conclusions

This study showed that ANC from berry wine and a variety of other phenolic compounds commonly present in fruits and vegetables had strong DPP-IV inhibitory effect *in vitro* and *in silico*. Computational docking analyses also showed for the first time that these natural phenolics could inhibit DPP-IV activity by binding tightly into the active sites of the enzyme. The biological activities, stability, and bioavailability of anthocyanins depend on their chemical structures. Blends were created to generate a mixture of potentially bioactive compounds commonly present in both blueberries and blackberries after fermentation, which can be optimized based on the characterization and potential benefit. 

Previous studies on wine compounds and biological activity indicated that it is not the presence of a single compound that is responsible for beneficial effects such as antioxidant capacity or ability to reduce inflammation, but rather involves several phenolic compounds. Major contributions are from compounds such as transresveratrol as well as minor contributions from cinnamic and hydroxycinnamic acids, cyanidin, and some phenolic acids [23]. The combination of these phenolic compounds within the blends produced from fermented blueberry and blackberry provided a unique potential for inhibition of DPP-IV. Therefore, while the inhibitory effects demonstrated by the anthocyanin-enriched blends are primarily due to the major anthocyanin components, the presence of other compounds also influenced the demonstrated potency.

In general, anthocyanins may protect beta-cells, increase the secretion of insulin, reduce the digestion of sugars in the small intestine, and thereby have multiple and simultaneous antidiabetic effects. Inhibitors of DPP-IV have been found to prevent pancreatic beta cell destruction in mice [24]. Extracts enriched in flavonoids have been seen to inhibit plasma DPP-IV [25]. 

The primary anthocyanin in the blackberry blends was delphinidin, which has previously shown potency to inhibit enzymatic activity of a glyoxalase I, which is being investigated as a target for prevention of cancer. Compared to other anthocyanins found in berries, (cyanidin and pelargonidin), delphinidin had the most potent DPP-IV inhibitory effect, suggesting the importance of interactions of the hydroxy groups on the B ring of anthocyanins. Further, binding modes indicated that the hydroxyl groups located at the R1 position greatly contribute to inhibitory potency and specificity to the binding site [26]. This previous study, along with the results from our research, indicates that the anthocyanin delphinidin can form several hydrogen bonds to several amino acids due to its hydroxyl groups at R1 position.

Our previous study also showed that the blueberry-blackberry wine contained high amounts of total anthocyanin [17]. However, correlation was not seen between DPP-IV inhibitory effect and anthocyanin concentration in ANC fractions (*P* > 0.05) from berry wines. For example, ANC3, ANC4, and ANC5 from blackberry wine were of similar IC_50_ values to inhibit DPP-IV, while the anthocyanin concentration was almost 4 times higher in ANC3 than in ANC4 and ANC5. Additionally, ANC4 and ANC5 had the same IC_50_ values and ANC concentration, but their anthocyanin compositions differed. These results indicate that delphinidin-3-arabinoside, as the major anthocyanin identified in blackberry blends, could contribute to the DPPIV inhibition, however, other ANC may also play an important role in DPP-IV inhibition. Therefore, DPP-IV inhibitory effect of ANC could depend on not only the concentration but the composition and structures of flavonoids present. More research should be conducted to clarify the relationship between DPP-IV inhibitory effect and anthocyanin structure of ANC from berries.

Phenolic compounds are widely recognized for their ability to improve diabetic conditions by decreasing blood glucose levels [27]. It is interesting that most of the ANC fractions showed potent DPP-IV inhibitory activity, with the lowest IC_50_ value from blackberry wine. Grape seed-derived procyanidins (GSPE) were also able to inhibit recombinant human DPP-IV activity, achieving around 70% inhibition at 200 mg/L of GSPE [12]. In order to compare with the GSPE, the percentage inhibition was given in this study as IC_50_ values. The concentrations of all ANC from blackberry wine for achieving the same inhibitory effect on DPP-IV were less than 200 mg/L. Especially for ANC3 from blackberry wine, the concentration of 41.9 mg/L could lead to around 70% inhibition of DPP-IV activity. These results suggest that ANC from blueberry and blackberry wine have strong DPP-IV inhibitory activity. The efficacy of the ANC to inhibit DPP-IV enzyme activity at a rate comparable to diprotin A and GSPE indicated that ANC may be able to act as naturally occurring DPP-IV inhibitors.

Many kinds of natural flavonoids exist in plants but only a few have been reported for DPP-IV inhibitory effect [12, 13]. In the present study of twenty-seven phenolic compounds commonly present in berries, citrus, soybeans, and other plant commodities, most flavonoids were determined to have DPP-IV inhibitory effect. It is interesting that most of the flavonoids tested in the present study showed lower IC_50_ values and therefore were more potent than the reference inhibitor standard diprotin A. Resveratrol, luteolin, apigenin and flavone showed the most potent DPP-IV inhibitory activity due to their lowest IC_50_ values. In particular, this study demonstrated that resveratrol was the most potent DPP-IV inhibitor with IC_50_ value at 0.6 nM exhibiting even lower values than sitagliptin (18 nM) and vildagliptin (3.5 nM) [10], two current pharmacologic drug inhibitors of DPP-IV. A summary of current foods and food components in the prevention of diabetes by Thomas and Pfeiffer [16] has indicated that the potential evidence for phenolic compounds is not conclusive; however, resveratrol was found to have a beneficial effect on protecting beta cells, which may be due to its ability to modulate the activity of DPP-IV.

DPP-IV has three binding pockets/active sites (S1, S2 and S3). The specificity pocket S1 is composed of the side chains of catalytic triad (Ser630, Asn710, and His740), which are involved in strong hydrophobic interactions [10]. The cavity near Glu205, Glu206 and Tyr662 residues is referred to as the S2 pocket. The S3 pocket of DPP-IV consists of Ser209, Arg358, and Phe357 [21]. The outside position of the S3 pocket in DPP-IV allows larger groups access to the site; on the other hand, the inside position of the S3 pocket favors smaller groups [28]. The four most potent compounds, resveratrol, luteolin, apigenin and flavone, had low *K*_*i*_ values to inhibit DPP-IV, which indicated that they had high affinity to the active sites of DPP-IV. The kinetic analysis showed that resveratrol and flavone inhibited DPP-IV activity in a competitive manner, while luteolin and apigenin were in a noncompetitive manner. Further computational docking analyses are consistent with the tested inhibitory manner of the phenolic compounds. Docking analysis showed that resveratrol and flavone bound well into all the three sites S1, S2 and S3 of DPP-IV, while luteolin and apigenin could only bind into S2 and S3 pockets. Although luteolin and apigenin could dock into S2 and S3 pockets, the kinetic analysis showed that they inhibited DPP-IV in a noncompetitive manner. We presume that the binding of luteolin and apigenin into S2 and S3 may lead to DPP-IV conformational changes, or changes in the side chain of amino acid residues of DPP-IV, and the catalytic activity will be decreased when the substrate is also bound.

We found that apigenin had a similar effect as resveratrol to directly inhibit DPP-IV activity, and genistein also exhibited a potent DPP-IV inhibitory effect. In the present study, most of the glycosylated flavonoids with two sugar groups, including naringin, rutin, narirutin, hesperidin, and neohesperidin, had no DPP-IV inhibitory effect. One explanation is that conjugation of bulky sugar groups to the flavonoid core structure could sterically hinder binding to the active sites within DPP-IV, thus resulting in no inhibitory capacity of the tested flavonoids. The computational docking analyses further supported this phenomenon. However, cyanidin-3-glucoside, which has been identified as the major ANC in different blackberry species [29], showed no statistical difference (*P* > 0.05) on DPP-IV inhibitory activity (IC_50_, 0.42 ± 0.09 *μ*M) than cyanidin (IC_50_, 1.31 ± 0.34) and malvidin (IC_50_, 0.74 ± 0.16). Considering ANC-enriched fractions from blueberry and blackberry wines contain a mixture of flavonoids with only one sugar group, flavonoids with monosugar groups may have better DPP-IV inhibitory effects than flavonoids with more sugar groups due to less steric hindrance. 

Flavone, luteolin, and apigenin have the same flavone core structure. However, flavone could dock into all three active sites of DPP-IV, while luteolin and apigenin could dock into only two of them. Computational docking showed comparably strong binding of luteolin and apigenin due to hydrogen bonds of ring B hydroxyls with residue Ser209 in the S3 pocket, for ring C 1′-oxygens with the NH of guanidine side chain of Arg358 in the S3 pocket, and for ring A 8′-hydroxyls with C=O groups of side chains of Glu361. These features also exist in the binding of other citrus flavonoids (including kaempferol, quercetin, hesperetin, and naringenin) to DPP-IV, which have the same flavone core structure. Even the binding of genistein, a soy isoflavone, to DPP-IV also had these features. Therefore, hydroxyls in these flavonoids are important to dock into active sites of DPP-IV with the same binding modes. Furthermore, the formation of *π*-interaction between A or B ring of citrus flavonoids and Arg669 or Arg358 also favors the binding of citrus flavonoids into S2 and S3 sites. Flavone has no hydroxyl residues capable of hydrogen bonding with residues in S2 and S3 pockets with the same binding modes as flavonoids like luteolin. Therefore, although it could dock into all the three pockets of DPP-IV, flavone had a higher *K*_*i*_ value due to absence of hydroxyl groups.

Significant correlations were seen between IC_50_ values of these flavonoids and their binding energies and *K*_*i*_ values determined computationally in the present study. In the docking studies, if a compound shows lower binding energy compared to the standard, it proves that the compound has higher activity [30]. These results indicated that more negative binding energy and smaller *K*_*i*_ result in tighter binding, and then more potent inhibitory effect. Meanwhile, a significant correlation also exists between the *K*_*i*_ values determined *in silico* and the number of hydroxyl groups of flavonoids (*r* = 0.56, *P* < 0.05), which indicates that more hydroxyl groups of flavonoids can result in higher inhibition constant and therefore higher IC_50_ value, indicating less affinity to bind the active site. This could explain why quercetin with five hydroxyls has a higher IC_50_ value (less potent) than the other citrus compounds, despite sharing the same flavone core structure. IC_50_ values of citrus compounds were also found to be significantly correlated with their numbers of hydroxyls.

We obtained *K*_*i*_ values using the computational analyses as well as experimentally. *K*_*i*_ values determined with the computational analyses were calculated from the binding energy. However, the binding energy is designed to score and rank conformations of ligand and protein and not designed to give accurate binding energy. Therefore, *K*_*i*_ values generated from autodock correlated with free binding energies significantly (*r* = 0.56, *P* < 0.05) but differed from the experimental *K*_*i*_ values.

In conclusion, our study demonstrated that ANC isolated from blueberry-blackberry wine blends and a variety of other phenolic compounds commonly present in citrus, berry, soy, and other plants could strongly inhibit DPP-IV activity. Resveratrol and flavone were competitive inhibitors which could dock into all the three active sites, while luteolin and apigenin bound to DPP-IV in a noncompetitive manner. Results obtained from this study further support the efficacy of flavonoids as naturally occurring DPP-IV inhibitors.

## Supplementary Material

Supplemental Information: Example of dose-response analysis of phenolic compounds on DPP-IV activity.

## Figures and Tables

**Table 1:** Anthocyanin (ANC) identification and quantification by HPLC at maximum absorption of 520 nm.

RT (min)	ANC ID	Anthocyanins (mg C3G equivalents/L) per blend (% Blueberry : % Blackberry)															
		100 : 0				75 : 25				25 : 75				0 : 100			
		ANC2	ANC3	ANC4	ANC5	ANC2	ANC3	ANC4	ANC5	ANC2	ANC3	ANC4	ANC5	ANC2	ANC3	ANC4	ANC5
24.44	Delphinidin-3-galactoside	6.3	10.8	10.0	nd	6.6	11.2	nd	nd	6.9	7.0	nd	nd	nd	nd	nd	nd
25.98	Delphinidin-3-glucoside	nd	26.8	**45.5**	nd	9.0	51.0	7.7	nd	12.3	17.7	nd	nd	8.8	nd	nd	nd
27.33	Cyanidin-3-galactoside	6.1	6.5	nd	nd	nd	6.3	nd	nd	17.7	nd	nd	6.5	23.8	nd	6.9	7.0
28.86	Delphinidin-3-arabinoside	nd	11.9	**30.9**	nd	nd	nd	nd	nd	**1079.3**	**716.1**	**30.1**	**16.8**	**1244.7**	**794.1**	**35.8**	**17.8**
29.69	Cyanidin-3-glucoside	6.4	23.4	nd	nd	60.6	**564.1**	29.7	9.3	10.2	12.2	nd	nd	10.1	6.5	6.1	nd
30.82	Cyanidin-3-arabinoside	nd	140.2	7.4	nd	11.0	16.7	8.0	7.2	nd	20.2	10.0	nd	116.8	nd	10.8	7.0
31.62	Petunidin-3-glucoside	9.7	7.3	24.9	10.5	56.1	79.6	13.3	6.7	**100.9**	nd	12.2	6.6	9.6	nd	12.2	7.2
32.69	Peonidin-3-glucoside	6.0	39.8	nd	nd	8.3	6.7	nd	8.0	8.4	nd	13.9	7.0	10.1	7.1	18.8	8.0
33.95	Petunidin-3-arabinoside	5.9	178.5	22.5	nd	nd	34.5	9.1	nd	14.3	8.3	10.4	6.5	9.7	nd	9.7	9.9
34.39	Malvidin-3-galactoside	74.8*	**292.1**	13.2	6.2	**266.1**	32.7	14.5	6.4	38.4	nd	15.5	nd	6.7	6.3	15.9	nd
35.19	Malvidin-3-glucoside	**63.1**	11.1	19.0	6.1	**266.3**	69.3	18.3	7.4	49.6	11.8	11.2	6.4	6.4	11.7	10.7	10.4
37.14	Malvidin-3-arabinoside	5.9	**278.9**	19.7	8.3	91.5	**127.2**	24.5	9.2	44.7	150.2	**30.6**	6.5	7.5	nd	**37.1**	7.0
39.03	Delphinidin-6-acetyl-3-glucoside	nd	7.3	17.9	6.7	6.3	12.6	11.4	6.4	nd	nd	nd	6.8	nd	7.2	nd	6.2
40.55	Cyanidin-6-acetyl-3-glucoside	nd	7.8	6.7	nd	8.9	nd	6.6	nd	6.1	nd	7.3	6.9	43.9	nd	nd	7.1
41.70	Malvidin-6-acetyl-galactoside	nd	26.7	7.1	nd	nd	13.6	nd	nd	7.7	nd	6.3	nd	nd	nd	7.2	6.6
42.25	Petunidin-6-acetyl-3-glucoside	nd	nd	12.9	6.0	nd	11.9	nd	nd	nd	nd	nd	nd	nd	nd	6.9	nd
44.40	Malvidin-6-acetyl-3-glucoside	nd	10.8	10.5	nd	11.5	19.4	8.4	nd	nd	8.9	8.1	6.4	nd	14.2	9.3	5.9

Sub-total ANC		190.5	1113.7	275.6	74.0	864.0	1112.3	183.8	81.6	1448.2	1073.3	239.2	146.8	1550.4	1204.8	302.8	229.8

Total ANC		1653.8				2241.7				2907.5				3267.8			

*Bold numbers indicate the dominant flavonoids in that particular ANC fraction.

nd: peak not detected.

**Table 2:** Anthocyanin (ANC) concentration (*μ*M) from blueberry-blackberry wine blends needed to inhibit DPP-IV enzyme activity by 50%^1,2^.

Blend ratio (% blueberry : % blackberry)	Fraction	IC_50 _(*μ*M)
100% Blueberry	ANC1	>300
	ANC2	4.67 ± 0.63^a^
	ANC3	0.64 ± 0.33^bc^
	ANC4	1.37 ± 0.58^abc^
	ANC5	0.72 ± 0.25^bc^

75% : 25%	ANC1	NA^3^
	ANC2	2.02 ± 0.56^ab^
	ANC3	0.41 ± 0.11^c^
	ANC4	0.22 ± 0.05^c^
	ANC5	0.36 ± 0.16^c^

25% : 75%	ANC1	NA
	ANC2	0.34 ± 0.10^c^
	ANC3	0.33 ± 0.08^c^
	ANC4	0.52 ± 0.18^c^
	ANC5	0.20 ± 0.10^c^

100% Blackberry	ANC1	NA
	ANC2	0.22 ± 0.03^c^
	ANC3	0.07 ± 0.02^c^
	ANC4	0.18 ± 0.07^c^
	ANC5	0.20 ± 0.09^c^

^1^IC_50_ values were determined from at least two independent duplicates done in triplicate and calculated in C3G equivalents. Values are means ± SEM. Means with different letters are significantly different (*P* < 0.05).

^2^The positive control of inhibition for DPP-IV was diprotin A (Ile-Pro-Ile) with an IC_50_ value of 4.21 ± 2.01 *μ*M.

^3^NA: No activity detected at >300 *μ*M.

**Table 3:** DPP-IV inhibition^1^ by flavonoids (IC_50_), their number of hydroxyl groups (OH), binding energy, inhibition constant (*K*_*i*_)^2^, H bonds involved, and *π* interactions.

Flavonoids	IC_50 _(*μ*M)	Number of OH groups	Binding energy (kcal/mol)	*K*_*i*_ (*μ*M)	H Bonds^3^	*π* interactions
Positive control Diprotin A	4.21 ± 2.01^bc^	0	−7.31	4.42	TYR547:HH-UNK:O22 UNK:H28-TYR666:OH UNK:H11-GLU206:OE1 UNK:H11-GLU206:OE2 UNK:H12-GLU205:OE2	*π*-cation TYR666-UNK:N13

Berry flavonoids Cyanidin	1.41 ± 0.25^e^	5	−5.95	43.43	TRP563:HN-UNK:O15 ALA564:HN-UNK:O18 UNK:H7-TYR48:OH UNK:H11-GLY741:O	*π*-*π* UNK-B:TRP629 UNK-B:TRP629 UNK-B:TRP629 UNK-B:TRP629

Cyanidin-3-glucoside	0.42 ± 0.09^ef^	8	−6.35	22.33	PHE357:HN-UNK:O20 ARG358:HH22-UNK:O17 ARG358:HE-UNK:O27 GLU361:HN-UNK:O9 ARG669:HH21-UNK:O31 UNK:H1-PHE208:O UNK:H6-GLU361:OE1 UNK:H20-GLU206:OE1 UNK:H20-UNK:O26	*π*-cation UNK-HIS126:NE2 UNK-ARG358:NH1 UNK-ARG358:NH2 UNK:ARG358:NH1

Malvidin	1.41 ± 0.44^ef^	3	−6.36	21.64	ARG356:HH11-UNK:O13 ARG356:HH11-UNK:O17 ARG358:HE-UNK:O11 ARG585:HH-UNK:O14 UNK:H6-ILE405:O UNK:H14-GLU206:O	*π*-*π* UNK-PHE357 UNK-PHE357 *π*-cation UNK-ARG669:NH2 UNK-ARG669:NH1 UNK-ARG669:NH2

Citrus flavonoids Luteolin	0.12 ± 0.01^f^	4	−6.26	25.83	ARG358:HE-UNK:O10 GLU361:NH-UNK:O1 UNK:H1-GLU361:OE1 UNK:H6-GLU205:O UNK:H7-SER209:OG UNK:H9-UNK:O7	*π*-cation UNK-ARG669:NH2 *π*-sigma UNK-PHE357:CB

Apigenin	0.14 ± 0.02^f^	3	−6.14	31.77	ARG356:HH11-UNK:O10 ARG358:HE-UNK:O5 GLU361:HN-UNK:O14 UNK:H4-UNK:O8 UNK:H5-GLU205:O UNK:H10-GLU205:O UNK:H10-SER209:OG	*π*-cation UNK-ARG669:NH2

Quercetin	2.92 ± 0.68^d^	5	−6.33	23.03	ARG356:HN-UNK:O5 ARG356:HN-UNK:O3 UNK:H2-UNK:O4 UNK:H3-ARG358:O UNK:H4-GLU206:O UNK:H5-SER209:OG	*π*-cation unk-ARG358:NH2 UNK-ARG358:NH2 UNK-ARG669:NH1 UNK-ARG669:NH2

Kaempferol	0.49 ± 0.02^ef^	4	−6.62	13.99	SER209:HG-UNK:O21 ARG356:HN-UNK:O8 PHE357:HN-UNK:O13 ARG358:HN-UNK:O13 UNK:H3-UNK:O8 UNK:H4-ARG358:O UNK:H5-GLU361:OE2 UNK:H10-SER209:OG	*π*-cation UNK-ARG356:NH1 UNK-ARG358:NH1 UNK-ARG358:NH2

Flavone	0.17 ± 0.01^f^	0	−6.64	13.57	No hydrogen bonds	No *π* interactions

Hesperetin	0.28 ± 0.07^ef^	3	−6.85	9.57	ARG358:HH22-UNK:O2 ARG669:HH21-UNK:O5 UNK:H2-GLU206:OE1 UNK:H1-UNK:O2 UNK:H3-ARG358:O	*π*-cation UNK-ARG358:NH1

Naringenin	0.24 ± 0.03^ef^	3	−6.83	9.90	ARG356:HH11-UNK:O20 ARG358:HE-UNK:O10 GLU361:HN-UNK:O19 UNK:H11-GLU361:OE1 UNK:H12-UNK:O11 UNK:H10-SER209:OG	*π*-cation UNK-arg358:NH1 UNK-ARG358:NH2

Eriocitrin	10.36 ± 0.09^a^	9	−9.07	225.96	ARG356:HH11-UNK:O8 PHE357:HN-UNK:O10 ARG358:HN-UNK:O10 ARG358:HE-UNK:O14 ARG429:HH22-UNK:O23 ARG669:HH21-UNK:O30 UNK:H5-ARG358:O UNK:H12-TYR585:OH UNK:H28-TYR585:OH UNK:H32-CYS551:O UNK:H20-GLU206:O UNK:H15-GLU206:O	*π*-cation UNK-ARG356:NH1

Soy isoflavone Genistein	0.48 ± 0.04^ef^	3	−6.5	17.31	ARG356:HN-UNK:O12 PHE357:HN-UNK:O10 ARG358:HN-UNK:O10 UNK:H4-ARG358:O UNK:H4-UNK:O10 UNK:H5-GLU36:OE1 UNK:H10-GLU206:O	*π*-*π* PHE357-UNK *π*-cation UNK-ARG356:NH1 UNK-ARG358:NH1 UNK-ARG358:NH1 UNK-ARG669:NH1

Grape stilbenoid Resveratrol	0.0006 ± 0.0004^g^	3	−6.54	15.96	ARG669:HH21-UNK:O7 UNK:H12-SER630:OG UNK:H5-SER209:OG UNK:H4-GLU206:O	

Other flavonoids EGCG	10.21 ± 0.75^a^	8	−4.39	604.91	GLN553:HN-UNK:O32 UNK:H8-GLU206:OE1 UNK:H11-TYR666:OH UNK:H15-TYR662:OH UNK:H15-TYR585:OH	*π*-*π* PHE357-UNK PHE357-UNK *π*-cation UNK-HIS740:NE2

Gallic acid	4.65 ± 0.99^b^	3	−3.96	1.25	ARG356:HH11-UNK:O11 PHE357:HN-UNK:O7 ARG358:HN-UNK:O7 TRY585:HH-UNK:O10 UNK:H3-ARG358:O UNK:H5-ILE405:O UNK:H6- ILE405:O	*π*-cation UNK-ARG356:NH2

Caffeic acid	3.37 ± 0.14^cd^	3	−5.23	147.66	ARG358:HE-UNK:O13 ARG358:HH22-UNK:O12 ARG669:HH21-UNK:O9 UNK1:H6-GLU206:OE1 UNK:H7-GLU206:OE1	*π*-*π* PHE357-UNK

^1^IC_50_ values were determined from at least two independent duplicates done in triplicate for each of the concentrations tested. Concentrations (*μ*M) were calculated based on the molecular mass of each pure compound. Values are means ± SEM. Means with different letters in each column are significantly different for DPP-IV (*P* < 0.05).

^2^ *K*_*i*_ values were obtained from computational docking as indicated in Materials and Methods section.

^3^UNK refers to phenolic compound or diprotin A.

## Abbreviations

ANC: Anthocyanins
C3G: Cyanidin-3-glucoside
DPP-IV: Dipeptidyl peptidase IV
GLP-1: Glucagon-like peptide-1
GIP: Glucose-dependent insulinotropic polypeptide
IC_50_: Concentration to inhibit 50% enzyme activity
*K*_*i*_: Enzyme inhibitor constant.
