# Supplementary material for: Berry and Citrus Phenolic Compounds Inhibit Dipeptidyl Peptidase IV: Implications in Diabetes Management
Source: Evid Based Complement Alternat Med. 2013 Aug 29;2013:479505. doi: 10.1155/2013/479505 (PMC3773436; doi:10.1155/2013/479505)

Supplemental information. Example of dose-response analysis of phenolic compounds on DPP-IV activity.

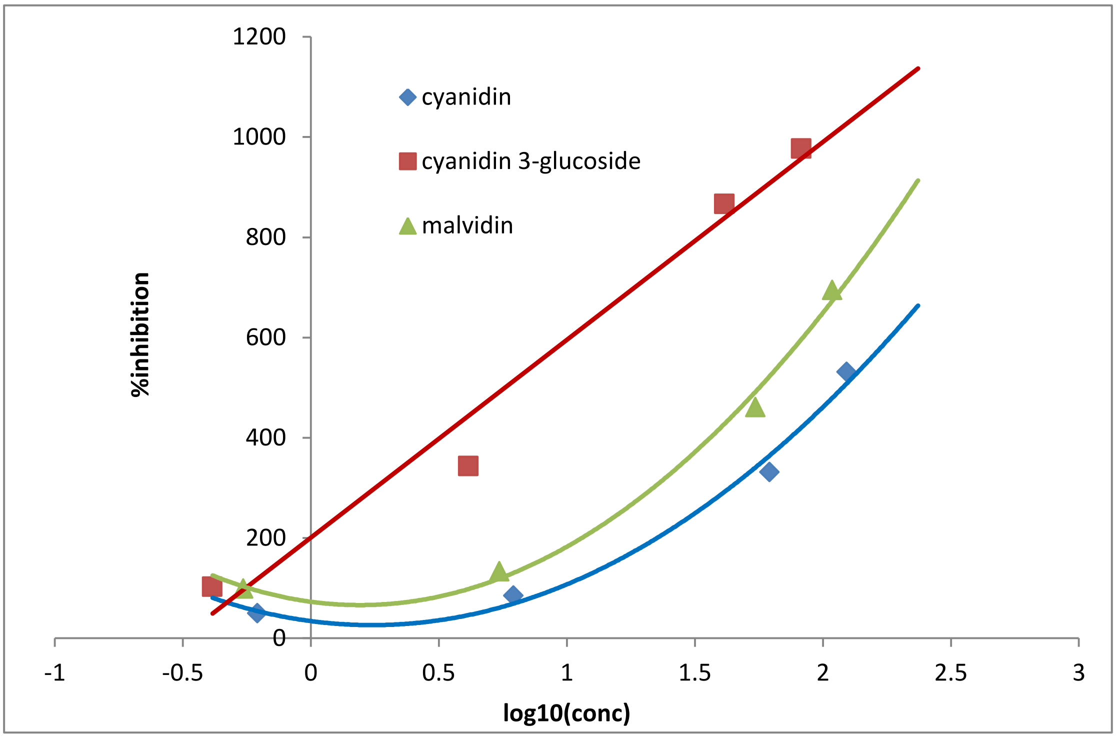

Supplement: Supplementary file 1 — Supplemental Information: Example of dose-response analysis of phenolic compounds on DPP-IV activity. [file 479505.f1.pdf]
